# Klippel Trenaunay Syndrome: A Case Report in an Adolescent Nigerian Boy

**DOI:** 10.3889/oamjms.2015.036

**Published:** 2015-03-28

**Authors:** Anthonia Asanye Ikpeme, Usang Edet Usang, Akan Wilson Inyang, Nchiewe Ani

**Affiliations:** 1 *University of Calabar Teaching Hospital, Radiology, University of Calabar, PMB 1115, Nigeria*; 2 *University of Calabar Teaching Hospital, Department of Surgery, University of Calabar, PMB 1115, Nigeria*

**Keywords:** Klippel-Trenaunay Weber syndrome, Adolescent, Port wine stain, infection

## Abstract

**AIM:**

This is to report a case of Klippel Trenauay Weber syndrome in a fifteen year old Nigerian boy. This is a rare syndrome and it is the first case to be reported in UCTH Calabar.

**CASE PRESENTATION:**

Product of a full term uneventful pregnancy, delivered to non-consanguineous apparently healthy parents. At birth was noted to gradually develop swelling on the right leg, worse at the right foot. There was crossed hemi-hypertrophy with right leg bigger than the left. As child grew symptoms worsened, parents separated and eventually he was abandoned to the streets. He presented at University of Calabar Teaching Hospital for medical care at the age of fifteen years with lymphatic obstruction, persistent foul smelling drainage, lipodermatosclerosis of right foot as well psycho-social and financial constraints. The diagnosis was made with x-rays and Doppler studies of the lower limb vessels. He is currently being managed conservatively with compression dressings on the affected limbs, Antibiotics for the infection and analgesics. De-bulking surgery is being anticipated at this time.

**CONCLUSION:**

This is a case of KTWS presenting in adolescence and due to its rarity in Nigeria, this report is to increase awareness.

## Introduction

Klippel Trenaunay Webber Syndrome (KTWS) is a rare congenital disorder characterized by asymmetric limb hypertrophy, usually of the lower limbs, as well as vascular anomalies and capillary malformations under the skin, termed the port wine stain [1]. These three main characteristics describe this syndrome but it could be associated with other anomalies like lymphatic obstruction, distal limb lipodermatosclerosis, affectation of the abdomino-pelvic vasculature leading to varying degrees of vascular malformations involving the gastro intestinal system, spleen, genito- urinary and central nervous system [2, 3]. This condition presents at birth and affects both males and females equally [4]. We present here a typical KTWS presenting at adolescence with overt complications in a Nigerian male. The aim is to make medical practitioners more aware of this rare condition and improve their diagnostic awareness.

## Case Report

**Figure 1:**
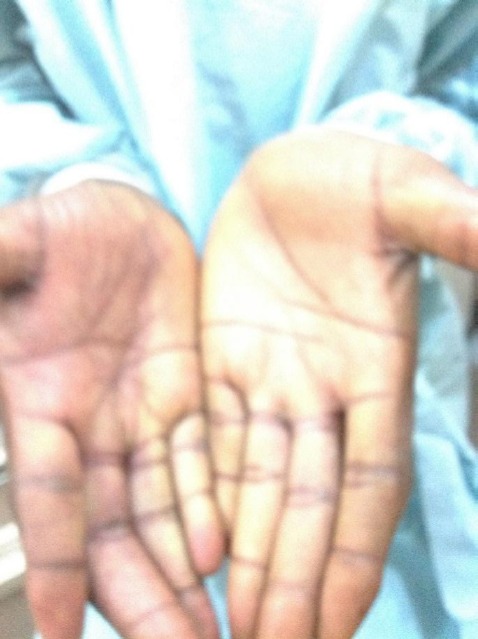
*Port wine stain noted on the right palm*.

M is a 15years old male who was delivered at home. Pregnancy and immediate post-partum period were uneventful. It was noted soon after birth that the right lower limb was progressively increasing in size when compared with the rest of the upper and lower limbs. He had an uneventful childhood except that he spent a lot of time at home and was withdrawn from other children. He was healthy but soon the limb began to be too heavy for him to move around with and he could no longer afford proper foot wears. His mother who raised him abandoned him which led him to the streets. He was soon recognized by a friend of his father and was rescued from the street. He presented at the University of Calabar Teaching Hospital for the first time at the age of 15 years. He was initially managed at the pediatric dermatologic clinic as a case of suspected elephantiasis and later referred to the Pediatric Surgery Unit where an initial diagnosis of congenital gigantism was made. He was referred for x-rays and Doppler studies of both lower limbs. The diagnosis of typical KTWS was made on the basis of clinical and radiological findings which included the following:

**Skin:** Port wine stains on both hands and feet (Fig. 1).

**Figure 2:**
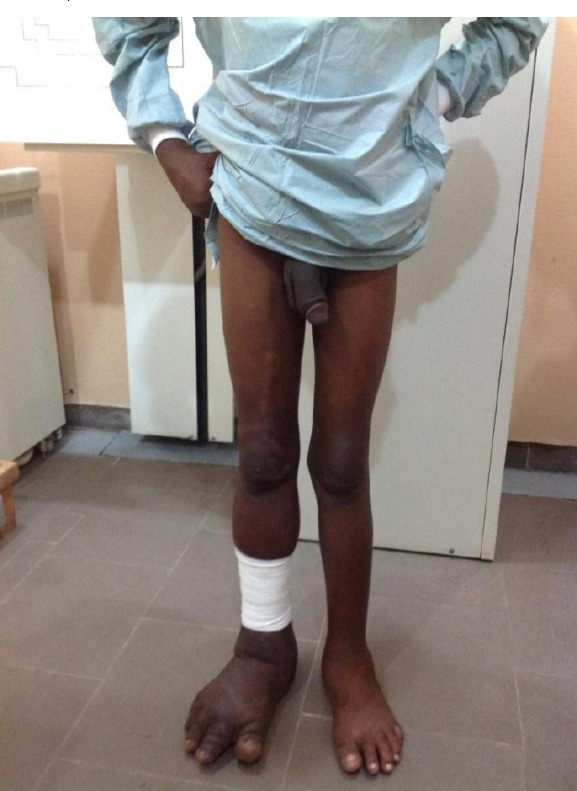
*Shows bilateral elongation of both lower limbs worse distally, and on the right with prominent superficial veins taking the course of the long saphenous vein with varicosities. Note also hypertrophied peni- scrotal organ*.

**Figure 3:**
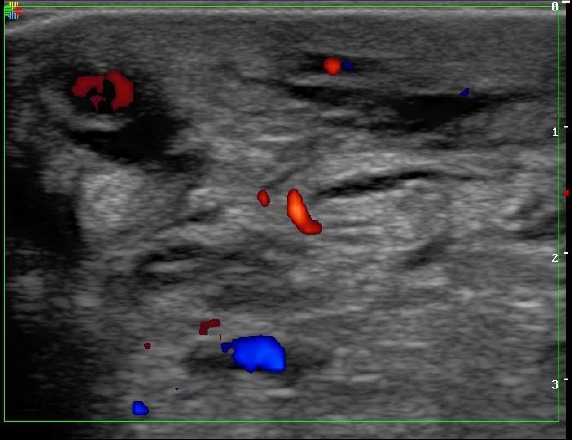
*2D Colour Doppler showing thickened soft tissue of the right foot with multiple hypervascular lakes-angiomas and obstructed lymphatic channels*.

**Musculo-skeletal system:** Marfan like hands and feet, no significant limb length discrepancy. There were marked differences in the circumferential dimensions of the lower limbs (Table 1). The right lower limb showed significant enlargement of the soft tissues of the leg and foot, worse distally, odematous right leg and foot as well as significant sclerosis of right foot with numerous hemangiomas (Fig. 2 and 3). There were no differences in circumferences of the upper limbs (mid-upper arm circumference 18.5 cm, mid-forearm circumference 18 cm.

**Table 1:** Shows circumferential dimension of both lower limbs.

	LAND MARK	RIGHT LOWER LIMB	LEFT LOWER LIMB
THIGH CIRCUMFERENCE	26 cm from ASIS	39.5 cm	37 cm
LEG CIRCUMFERENCE	27 cm from knee joint line	32 cm	25 cm
FOOT CIRCUMFERENCE	12 cm from ankle joint	37 cm	27 cm

**Cardiovascular system:** Significant right lower limb varicosities, multiple sinuses in which clear but foul smelling lymph was noted to be draining (Fig. 2).

**Genitourinary System:** enlarged peni-scrotal organ with subcutaneous oedema (Fig. 2).

All other systems were essentially normal. Patient in addition was asked to carry out multi detector computerized angiography which has not been done due to financial constraint.

Firm bandaging of the affected limb was applied in order to reduce lymphatic flow and prevention of infection. Antibiotics and pain relief were also prescribed. Patient is still being awaited as the managing team have decided to bear the cost of the rest of his investigations and treatment. Surgical debulking of the right foot is being envisaged at the moment.

## Discussion

Klippel Trenaunay Weber Syndrome is a rare congenital condition in which blood vessels and/or lymphatic vessels fail to form properly [1]. The three main features that describe this condition are a port wine stain or naevi which is caused by capillary malformations that create a reddish-purplish discoloration of the skin, vascular anomalies and hypertrophy of the affected limb bones and soft tissues [1, 4-6]. It was first reported in 1900 by two French physicians - Maurice Klippel and Paul Trenaunay, who described two patients who had a triad of port wine stain, varicosities of an extremity and hypertrophy of the affected limb bones and soft tissues [3, 6]. Few years later Parks Weber independently described a syndrome which include the above stated triad and the presence of an arterio-venous malformation in the affected limb [7, 8].

Currently, while some authors insist the syndromes be separated, others believe they should be described together and termed KTWS [5, 6]. The index patient had not undergone an angiography therefore it was difficult to classify it as KTWS. However mortality is said to be significantly higher in KTWS and the patient appeared quite healthy with no suggestion of an arterio-venous malformation clinically, though there were few findings on Doppler that suggested possible small arterio - venous fistulae in the right thigh. There are two types of KTWS - typical and atypical [4]. Typical KTWS always includes a port wine stain while atypical type does not include a port wine stain [4]. The latter is quite rare. The index patient had port wine stains on both palms and soles of both feet, seen as a pink discolouration of one half of the palm/foot.

The cause is unknown; however a few theories have been postulated. Popular among them is that of Baskerville et al. [4] that states that a mesodermal defect during embryogenesis causes maintenance of microscopic arteriovenous communications resulting in KTWS. Whelan et al. [9] encountered a balanced 5:11 translocation (an equal exchange of material between two chromosomes with no genetic information added or lost). There is neither gender nor racial predilection [4, 5]. Diagnosis usually made *in utero* or at birth [4, 10]. The index patient presented first for medi-care at the age of fifteen.

Diagnosis is made by ultrasound, Doppler studies, multi-detector CT scan, conventional arteriography and Magnetic Resonance Angiographic studies which show the types of vascular anomalies that cannot be seen on conventional ultrasound and x-rays [8, 11]. X-rays show hypertrophy involving bones with delayed epiphyseal fusion [8].

The index patient showed all four limbs to be elongated nearly symmetrically with no evidence of epiphyseal fusion. No calcified phleboliths were seen in any of the x-rays though this is a recognized x-ray finding [7, 10].

Doppler scan revealed prominent varicosities especially of the superficial veins, haemangiomas within the foot, engorged lymphatics, significant subcutaneous oedema and thickening of the right foot, termed lipodermatosclerosis [8]. An abdominal scan done showed no abnormalities. Many intra-abdominal malformations have been recognized [2, 3, 11] and therefore a scan of the liver, spleen, kidneys and the abdominal aorta is absolutely necessary.

This is the first reported case in Nigeria to the best of the knowledge of the authors. KTWS occurs mostly sporadically and just over 1000 cases have been reported till date [12].

A multi detector computerized tomographic or magnetic resonance angiography was advised for the index patient but this was not done due to financial constraints. In our environment out of pocket expenses is the main modality of payment for health care services. Angiographic findings that maybe seen, are lower leg superficial varicoid drainage without a deep venous system. The marginal vein of Servelle is a pathognomic finding [12]. This was however not apparent in the index patient either clinically or on Doppler scan. The differential diagnosis of the condition include Proteus syndrome, a rare overgrowth syndrome and can only be diagnosed if there is cerebriform cutaneous neavus, adipose tissue deposition, tumors and vascular anomalies [13]. Others are CLOVE syndrome; also overgrowth of body tissues including the paraspinal and chest regions as well as lymphatic and capillary malformations. The above two are not characterized by port wine stain, which the index patient had.

Maffucci’s syndrome and Sturge Webber’s, present with port wine stain but show no hypertrophy of other body tissues [14]. The combination of the aforementioned clinical and radiological features strongly supports the diagnosis of Klippel Trenaunay Webber syndrome.

In conclusion, KTWS is a rare condition but appears to be seen in all parts of the world. This is the first report from Nigeria. It can be easily misdiagnosed as demonstrated in the present case. Clinicians should be kept aware of the condition.
